# Phenolic Profile and Bioactive Properties of *Carissa macrocarpa* (Eckl.) A.DC.: An In Vitro Comparative Study between Leaves, Stems, and Flowers

**DOI:** 10.3390/molecules24091696

**Published:** 2019-04-30

**Authors:** Fedia Souilem, Maria Inês Dias, Lillian Barros, Ricardo C. Calhelha, Maria José Alves, Fethia Harzallah-Skhiri, Isabel C.F.R. Ferreira

**Affiliations:** 1 Centro de Investigação de Montanha (CIMO), Instituto Politécnico de Bragança, Campus de Santa Apolónia, 5300-253 Bragança, Portugal; souilemfedia@gmail.com (F.S.); maria.ines@ipb.pt (M.I.D.); lillian@ipb.pt (L.B.); calhelha@ipb.pt (R.C.C.); maria.alves@ipb.pt (M.J.A.); 2 Laboratoire de Recherche “Bioressources”: Biologie Intégrative & Valorisation (BIOLIVAL) LR14ES06, Institut Supérieur de Biotechnologie de Monastir, Avenue Tahar Hadded, BP 74,5000, Université de Monastir, Monastir 5000, Tunisia; fethiaprosopis@yahoo.fr

**Keywords:** *Carissa macrocarpa*, plant aerial parts, phenolic profile, bioactivities, Pearson’s correlation

## Abstract

The present work aimed to characterize leaves, stems, and flowers of *Carissa macrocarpa* (Eckl.) A.DC., by performing an analysis of the phenolic compounds by HPLC-DAD/ESI-MS, correlating them with bioactive properties, such as antioxidant, anti-inflammatory, cytotoxic, and antimicrobial activities. Thirty polyphenols were identified in the hydroethanolic extract, including phenolic acids, flavan-3-ols, and flavonol glycosides derivatives (which presented the highest number of identified compounds). However, flavan-3-ols showed the highest concentration in stems (mainly owing to the presence of dimers, trimmers, and tetramers of type B (epi)catechin). Leaves were distinguished by their high antioxidant and anti-inflammatory activities, as well as their bactericidal effect against *E. coli*, while stems presented a higher cytotoxic activity and bactericidal effect against Gram-positive bacteria. Moreover, a high correlation between the studied bioactivities and the presence of phenolic compounds was also verified. The obtained results bring added value to the studied plant species.

## 1. Introduction

The genus *Carissa* belongs to the family of *Apocynaceae*, which normally has a high content of phenolic compounds, such as flavonoids, as well as lignans and sesquiterpenes. Accordingly, this plant genus presents several therapeutic applications such as antioxidant, analgesic, anti-inflammatory, hypolipidemic, wound healing, antimicrobial, antidiabetic, antiepileptic, anti-cancer, diuretic, hepatoprotective, and improvement of nephrotoxicity [1].

*Carissa macrocarpa* (Eckl.) A.DC. (syn: *C. grandiflora* (E.Mey.) A.DC.) is a native plant from South Africa, KwaZulu-Natal, commonly known as Natal plum [2,3]. It is an ornamental shrub, characterized by large, green, lush, and persistent leaves; star-shaped and white flowers; and edible oval fruits [3,4]. The ripe fruits are delicious and can be used for the preparation of jams, sauces, desserts, yogurt, jellies, and ice cream, while this plant is also used in traditional medicine for the treatment of diarrhea in livestock, cough, and venereal diseases [5,6]. *C. macrocarpa* leaves, stems, and roots present antioxidant and antimicrobial activities [3,4], and the leaves present cytotoxic activity [7], owing to the presence of various secondary metabolites, such as flavonoids, saponins, triterpenoids, anthraquinones, and tannins 

The presence of different phenolic compounds, such as flavonoids, hydroxycinnamic and hydroxybenzoic acids, coumarins, xanthones, chalcones, stilbenes, lignins, and lignans have been described in numerous publications regarding the phenolic profile of the genus *Carissa*. These compounds are known for their important role in cancer treatment [8], hepatoprotective effects [9], and antifungal properties [10]. Some of these biological properties are directly related to the antioxidant activity of these compounds, in accordance with that described by Martins et al. [10]. 

To the author’s best knowledge, there are no previous studies describing the anti-inflammatory activity of *C. macrocarpa* leaves, stems, or flowers, and only few studies could be found regarding antioxidant, cytotoxic, and antibacterial properties of different extracts obtained from leaves and stems. Moreover, there is no report on *C. macrocarpa* flowers. Therefore, the aim of the present study was to established the individual phenolic profile of the hydroethanolic extracts obtained from leaves, stems, and flowers of *C. macrocarpa*, being further correlated with their antioxidant, antibacterial, anti-inflammatory, and cytotoxic properties.

## 2. Results and Discussion

### 2.1. Phenolic Profile of the Aerial Parts of C. Macrocarpa

**Figure 1:**
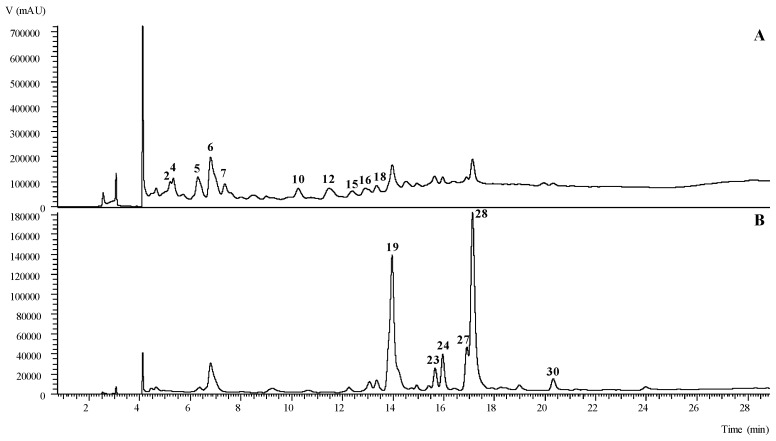
Phenolic profile of the hydroethanolic extract of *C. macrocarpa* leaves recorded at 280 nm (**A**) and 370 nm (**B**) obtained by HPLC-DAD/ESI-MS.

Table 1 presents the chromatographic characteristics (obtained by HPLC-DAD/ESI-MS) and tentative identification of the phenolic compounds present in the hydroethanolic extracts obtained from leaves, stems, and flowers of *C. macrocarpa*. The quantification of each identified compound is presented in Table 2. An exemplificative phenolic profile of the hydroethanolic extract prepared from leaves is presented in Figure 1.

Thirty phenolic compounds were tentatively identified in the hydroethanolic extracts prepared from *C. macrocarpa* leaves, stems, and flowers: nine phenolic acids (chlorogenic, coumaric, and syringic coumaric acid derivatives), thirteen flavonols (kaempherol and quercetin derivatives), and eight flavan-3-ols ((epi)-catechin derivatives). To the author’s best knowledge, all the phenolic compounds were tentatively identified for the first time in the hydroethanolic extracts of *C. macrocarpa.*

Phenolic acids were found in higher amounts in the hydroethanolic extract of flowers, and in very close amounts to flavanols in the same extract, while leaves showed the lowest amount of phenolic acids. Four chlorogenic acid derivatives (peaks **1**, **5**, **6**, and **8**), four *p*-coumaric acid derivatives (peaks **2**, **4**, **13**, and **15**), and one syringic acid derivative (peak **3**) were tentatively identified. Peaks **1**, **5**, **6**, and **8** ([M − H]^−^ at *m*/*z* 353) were tentatively identified as 3-*O*-caffeoylquinic acid (peak **1**), 4-*O*-caffeoylquinic acid (*cis* and *trans*, peak **5** and **6**, respectively), and 5-*O*-caffeoylquinic acid (peak **8**). The assignments of the different caffeoylquinic acid isomers were made using the hierarchical key system previously reported by Clifford et al. (2003) and Clifford et al. (2005) [11,12]. Moreover, 5-*O*-caffeoylquinic acid (peak **8**) was positively identified in comparison with the available commercial standard. Peaks **2**, **4**, **13**, and **15** ([M − H]^−^ at *m*/*z* 337) were tentatively identified according to their MS^2^ fragmentation as *cis* and *trans* 3-*p*-coumaroylquinic acid, and *cis* and *trans* 5-*p*-coumaroylquinic acid, respectively, as previously reported by Clifford et al. (2003) and Clifford et al. (2005) [11,12]. The identification of the compounds 2/4, 5/6, and 13/15 as *cis*/*trans* isomers, was based on experimental results previously published by our research group [13], also following the information described by Clifford and coworkers [14,15] regarding these isomers. The hydroxycinnamoyl *cis* and *trans* derivatives were distinguished, after UV irradiation (366 nm, 24 h) of these acids in our laboratory [13]. Compound **3** with MS^2^ fragments at *m*/*z* 197 ([syringic acid-H]^−^) resulting from the loss of a hexosyl moiety (−162 u) was tentatively assigned as syringic acid hexoside.

Flavonol glycoside derivatives of quercetin (MS^2^ fragment at *m*/*z* 301) and kaempferol (MS^2^ fragment at *m*/*z* 285) were the main molecules present in this family of phenolic compounds. Quercetin-3-*O*-rutinoside (rutin; peak **28**) and kaempferol-3-*O*-rutinoside (peak **30**) were identified upon comparison of their chromatographic characteristics with available commercial standards. These compounds have already been identified in the Apocyneaceae family [16].

Peaks **18**, **21**, **25**, and **26** ([M − H]^−^ at *m*/*z* 739) and **20** ([M − H]^−^ at *m*/*z* 755) provided the same consequently fragmentation losses of a deoxyhexosyl unit (146 u) and deoxyhexosyl-hexosyl unit (308 u), indicating the location of each residue on different positions of the aglycones of kaempferol and quercetin, respectively, being tentatively identified as kaempferol-*O*-deoxyhexoside-*O*-deoxyhexosyl-hexoside isomers 1, 2 3, and 4, and quercetin-*O*-deoxyhexoside-*O*-deoxyhexosyl-hexoside, respectively. Peak **23** also showed a pseudomolecular ion [M − H]^−^ at *m*/*z* 739, presenting a unique MS^2^ fragment at *m*/*z* 285 (−454 u, which corresponded to 146 + 146 u + 162 u) indicating that the three sugar units were linked together in the same oxygen position of the aglycone, and in this particular case, kaempferol, being tentatively identified as kaempferol-*O*-di-deoxyhexosyl- hexoside. Peak **24** presented a pseudomolecular ion [M − H]^−^ at *m*/*z* 575, releasing an MS^2^ fragment at *m*/*z* 285, corresponding to the loss of acetyl (42 u), malonyl (86 u), and hexosyl (162 u) residues, being tentatively identified as acetylkaempherol-*O*-malonylhexoside. Peaks **22** and **27** ([M − H]^−^ at *m*/*z* 609; MS^2^ at *m*/*z* 301) and **29** ([M − H]^−^ at *m*/*z* 593; at *m*/*z* 301) showed a single MS^2^ fragment that indicated the loss of 308 u, corresponding to two sugar units linked together (deoxyhexosyl-hexoside moieties), being tentatively identified as quercetin-*O*-deoxyhexosyl-hexoside isomer 1 and 2, and kaempherol-*O*-deoxyhexosyl-hexoside, respectively. Peak **10** ([M − H]^−^ at *m*/*z* 755) fragmented at *m*/*z* 593 (−162 u, hexosyl unit) and 285 (−308 u, deoxyhexosyl-hexoside unit), being tentatively identified as kaempherol-*O*-hexoside-*O*-deoxyhexosyl-hexoside.

Flavan-3-ols were the main family of phenolic compounds present in the hydroethanolic extracts of stems of *C. macrocarpa*; not being detected in the flower samples. Peaks **7**, **9**, **11**, **12**, **14**, **16**, **17**, and **19** were tentatively identified based on their chromatographic characteristics (pseudomolecular analysis and MS^2^ fragmentation pattern) coherent with B-type (epi)catechin derivatives. Thus, they were tentatively identified as B-type (epi)catechin dimers (peaks **7** and **9**, [M − H]^−^ at *m*/*z* 577), trimers (peaks **11** and **16**, [M − H]^−^ at *m*/*z* 865), and tetramers (peaks **12**, **14**, and **19**, [M − H]^−^ at *m*/*z* 1153) [17]. Peak **17** was tentatively identified based on the information found in the literature as a (epi)catechin trimer with a type-A linkage, thus being assigned as an A-type (epi)catechin trimer [18].

The phenolic profile of the three plant parts was relatively similar; nevertheless, they all presented different major compounds. For the leaves, *cis* 5-*p*-coumaroylquinic acid (peak **13**, 2.9 mg/g extract) was the major molecule, while *trans* 5-*p*-coumaroylquinic acid (peak **15**, 7.3 mg/g extract) was the major compound found in stems, and *cis* 4-*O*-caffeolyquinic acid (peak **5**, 10.35 μg/g extract) was the main compound found in flowers.

### 2.2. Bioactivities of the Hydroethanolic Extracts of Leaves, Stems, and Flowers

Results regarding in vitro antioxidant activity of the ethanol/water (80:20 *v*/*v*) extracts prepared from leaves, stems, and flowers of *C. macrocarpa* are described in Table 3.

Overall, all samples presented high antioxidant activity, but the statistical analysis did not show significant differences that could allow to particularize a more potent plant part. However, observing the four used methodologies, the leaves presented the lowest EC_50_ values for DPPH scavenging activity, reducing power, and β-carotene bleaching inhibition (EC_50_ = 26 ± 1, 36 ± 1, and 300 ± 1 μg/mL, respectively), while for the TBARS assay, the stems showed the lowest EC_50_ value (12.1 ± 0.1 μg/mL). The antioxidant activity of methanolic extracts from leaves and stems of *C. grandiflora* originated in Pakistan was previously reported, presenting similar results for DPPH scavenging activity of leaves (0.2089 ± mg/mL), but lower results for stems (0.0615 mg/mL) [4]. The cytotoxic effects of the hydroethanolic extracts prepared from *C. macrocarpa* were evaluated on non-tumour (PLP2) and four human tumour cell lines, with the results being summarized in Table 3. It can be observed that all samples exhibited anti-proliferative activity on the four tested tumour cell lines with GI_50_ values ranging between 52.1 ± 0.3 and 167 ± 2 μg/mL, with the exception of the hydroethanolic extract of the flowers against HepG2. Unlike the antioxidant activity, the stems showed the lowest GI_50_ values in all the cell lines studied. It is worthwhile mentioning that any of the samples exhibited toxicity against the normal liver cell line (PLP2), up to the maximal tested concentration (GI_50_ > 400 μg/mL). Khatun et al. (2017) [19] investigated *C. macrocarpa* methanolic extract, which reduced the viability of adenocarcinoma cell lines (SW-480 and SW-48) with IC_50_ values of 140.6 and 376.6 μg/mL, respectively, after 24 h of treatment, and 108.4 and 290.0 μg/mL, respectively, after 48 h of treatment. Sehar et al. (2011) [20] studied the aqueous extract of *C. spinarum* stems and its *n*-butanol fraction, which exhibited a potential cytotoxic effect on a wide range of human tumour cell lines, with apoptotic activity in human leukaemia HL-60 cells, through the mitochondrial dependent pathway in HL-60 cells. In addition, the methanolic extract of *Carissa opaca* Stapf ex Haines leaves and their fractions were tested against MCF-7 breast cancer cell line, indicating that the fractions were more active than the crude extracts [21]. Carandinol extracted from the leaves of *C. carandas* exhibited significant in vitro cytotoxicity against HeLa, PC-3, and 3T3 cell lines [22]. Lignans, carissanol, carinol, and nortrachelogenin extracted from the stems of *C. spinarum* displayed cytotoxicity against breast (MCF-7) and lung (A549) tumour cell lines [23]. 

The results regarding the anti-inflammatory activity of *C. macrocarpa* hydroethanolic extracts are also summarized in Table 3. All the samples revealed inhibition of the NO production with higher GI_50_ values in comparison with the positive control, and it can be verified that the highest activity was observed in the leaves (GI_50_ = 179 ± 6 μg/mL). To the author’s best knowledge, there are no previous reports regarding the anti-inflammatory activity of *C. macrocarpa*. However, the anti-inflammatory activity of naringin isolate from the leaves of *C. carandas* was investigated in vivo by a carrageenan induced hind rat pawedema model and in vitro by measuring its inhibitory effect on LPS induced release of NO from RAW 264.7 macrophages. The results showed that naringin (compound not found in our samples) exhibited potent inhibition of inflammation and inhibited LPS induced release of NO from macrophages (IC_50_ = 6.4 μM) [24].

The hydroethanolic extracts of leaves, stems, and flowers of *C. macrocarpa* were tested for their antimicrobial activity against selected clinical isolates, representing both Gram-positive and Gram-negative bacteria. The results of the obtained minimum inhibitory concentration (MIC) values of the hydroethanolic extracts of *C. macrocarpa* are presented in Table 3. Gram-positive bacteria were more sensitive to the extracts presenting lower MIC values ranging from 0.625 to 20 mg/mL, and were more susceptible to both leaves and stems. The best results among Gram-negative bacteria were observed for *Escherichia coli* ESBL and *Morganella morganii*. Regarding *Klebsiella pneumoniae* and *Klebsiella pneumoniae* ESBL, the extracts did not express any activity up to the maximal tested concentration. The antibacterial activity of *C. macrocarpa* extracts is supported by the studies performed by Abbas et al. (2014) [4], which reported a higher MIC value in the methanolic extracts of *C. macrocarpa* stems and leaves against three pathogenic microorganisms (*E. coli*, *Staphylococcus aureus*, and *Staphylococcus epidermidis*), with MIC values ranging between 0.39 and 1.88 mg/mL.

Table 3 also presents the Pearson’s correlation analysis between the bioactivities and the sum of total phenolic acid derivatives, total flavan-3-ols, total flavonols, and total phenolic compounds. The results with a confidence level below 70% are not shown in the table, being classified as a moderate correlation (confidence level between 50% and 70%) and weak and negligible correlations (confidence level between 50% and 30% and between 30% and 0%, respectively). Regarding antioxidant activity, it can be observed that flavan-3-ols had the highest correlations with β-carotene bleaching inhibition and TBARS assay (*r*^2^ = 0781, and 0794, respectively), while DPPH scavenging activity presented a higher correlation to the phenolic acids (*r*^2^ = 0.887), and reducing power to the total phenolic compounds (*r*^2^ = 0.995). The antioxidant potential of flavan-3-ols derivatives has been widely reported [25]; however, because of the structural diversity of these type of compounds, their in vivo activity can be very different, which is the reason they did not correlate so strongly with the biochemical assay of β-carotene bleaching inhibition and TBARS. Regarding the cytotoxic activity, phenolic acids showed correlations with all the studied cell lines, although showing a higher correlation with MCF-7 and NCI-H460 (*r*^2^ = 0.862 and 0.911, respectively). For HeLa and HepG2 cell lines, the highest correlation levels were observed with total phenolic compounds (*r*^2^ = 0.781) and flavonols (*r*^2^ = 0.953), respectively. Finally, the correlations on the antimicrobial activity revealed that it is with Gram-negative bacteria that phenolic compounds correlate more, with values that ranged between *r*^2^ = 0.720 and 0.938. However, the results obtained for *E. coli* showed a correlation level of *r*^2^ = 0.961 and *r*^2^ = 0.952 with the phenolic acids and flavonols, respectively, a much higher correlation than those observed for the other Gram-negative bacteria. In the groups of phenolic acids and flavonols, no compound stood out and, therefore, perhaps synergetic effects of all compounds are responsible for the high correlations observed. However, the biological properties of hydroxycinnamic acids and quercetin and kaempferol derivatives have already been abundantly observed and described; notwithstanding, the synergies that occur in natural extracts may favour the biological properties.

## 3. Materials and Methods

### 3.1. Plant Material and Preparation of the Hydroethanolic Extracts

The samples of *Carissa macrocarpa* (Eckl.) A.DC. (leaves, stems, and flowers) were collected in Monastir, Tunisia during 2016. The samples were dried until at a constant weight in an incubator at 35 °C. Then, the plant material was ground to approximately 40 mesh, and the homogeneous samples were stored in a desiccator protected from light.

The hydroalcoholic extract was obtained by maceration using aqueous ethanolic solution (80%, *v*/*v*; 30 g/mL) as the extraction solvent, applying the previous conditions reported by the authors (Barros et al., 2013). After filtration (Whatman n°4 filter), the solvent was first evaporated at 40 °C, under reduced pressure, in a rotary evaporator (Büchi R-210, Flawil, Switzerland) and the residual solvent was removed in a freeze drier (−49 °C, 0.089 bar, during 48 h, FreeZone 4.5, Labconco, Kansas City, MO, USA).

### 3.2. Phenolic Profile of the Hydroethanolic Extracts of Leaves, Stems, and Flowers

The dry extracts were re-suspended at a concentration of 5 mg/mL using aqueous ethanol (80%, *v*/*v*) and filtered (0.2 µm disposable LC filter disk, 30 mm, nylon). Afterwards, the phenolic profile was found by liquid chromatography with a diode-array detector (280, 330, and 370 nm wavelengths) coupled to an electrospray ionization mass spectrometry operating in negative mode (Dionex Ultimate 3000 UPLC and Linear Ion Trap LTQ XL, Thermo Scientific, San Jose, CA, USA), as previously described by the authors [26]. The phenolic compounds were identified according to their chromatographic characteristics by comparison with those obtained with standard compounds and with literature. Calibration curves of appropriate standards were obtained in the range of 200–5 µg/mL for the quantitative analysis. The results were expressed in mg per g of extract (mg/g).

### 3.3. Bioactivities of the Hydroethanolic Extracts of Leaves, Stems, and Flowers

#### 3.3.1. Antioxidant Activity

The extracts were diluted in distilled water at a concentration of 10 mg/mL; then, successive dilutions were carried out (5000 and 6.25 µg/mL). The DPPH radical-scavenging activity, reducing power, inhibition of β-carotene bleaching, and TBARS assay were the methodologies applied to determine the antioxidant activity [27]. The results were expressed as EC_50_ values (sample concentration providing 50% of antioxidant activity) and Trolox was used as a positive control.

#### 3.3.2. Cytotoxic Activity

The extracts were re-dissolved in water at a 8 mg/mL concentration and further diluted in the range of 400 to 6.25 µg/mL. The cytotoxic properties were evaluated using four human tumor cell lines: MCF-7 (breast adenocarcinoma), NCI-H460 (non-small cell lung cancer), HeLa (cervical carcinoma), and HepG2 (hepatocellular carcinoma). A non-tumor cell line (PLP2) was also evaluated using a procedure previously described in Abreu et al. (2011) [28]. Sulforhodamine B assay was carried out [29], with Ellipticine used as positive control, and a negative control was provided by each suspension of cells. The results were expressed in GI_50_ values (concentration that inhibited 50% of the cell proliferation).

#### 3.3.3. Anti-Inflammatory Activity

The extracts were re-dissolved in water at a concentration of 8 mg/mL and then diluted in the range of 400 to 6.25 µg/mL. A mouse macrophage-like cell line RAW 264.7 was used in this study and the Griess Reagent System (GRS) kit was applied to determine the nitric oxide, measured at 515 nm (ELx800 microplate reader, Bio-Tek Instruments, Inc; Winooski, VT, USA), as described previously [30]. The results were expressed in EC_50_ values (sample concentration providing 50% of inhibition of NO production) and Dexamethasone was used as a positive control, while in negative controls, no LPS was added. 

#### 3.3.4. Antibacterial Activity

The extracts were re-dissolved in water to obtain a stock solution of 100 mg/mL and then diluted in the range of 20 to 1.25 µg/mL. The antimicrobial potential of the extracts was assessed using five Gram-negative bacteria and three Gram-positive bacteria. For each bacteria, the minimum inhibitory concentration (MIC) and minimum bactericidal concentration (MBC) were determined using a colorimetric assay, as described by Svobodova et al. (2017) [31].

### 3.4. Statistical Analysis

Triplicates of the samples were assayed and three repetitions of each methodology were performed, with the results being expressed as mean values and standard deviations (SD). The significant differences between samples were evaluated using one-way analysis of variance (ANOVA) followed by Tukey’s HSD Test with *p* = 0.05 (SPSS v. 23.0 program); when there were less than three samples, a Student´s *t*-test was applied (*p* = 0.05). Furthermore, a Pearson’s correlation analysis between the bioactivities and all the sum contents of the analysed compounds (total phenolic acid derivatives, total flavan-3-ols, total flavonols, and total phenolic compounds) was carried out, with a 95% confidence level.

## 4. Conclusions

The results obtained showed that the different parts of *C. macrocarpa* can be used as sources of phenolic compounds, with high bioactive potential to be exploited in the development of novel pharmaceutical formulations, for example. Overall, among the thirty phenolic compounds identified in the hydroethanolic extracts prepared from leaves, stems, and flowers of *C. macrocarp*, three distinct families were found: phenolic acids, flavan-3-ols, and flavonols. The last-mentioned group presented the highest number of identified compounds; thus, flavan-3-ols showed the highest concentration in stems (mainly owing to the presence of dimers, trimmers, and tetramers of (epi)-catechin). The phenolic acids were found in higher amounts in flowers, mainly owing to the presence of 4-*O*-caffeoylquinic acid. Leaves were distinguished by their high antioxidant and anti-inflammatory activity, as well as bactericidal activity against *E. coli*. Stems presented a high cytotoxic activity and bactericidal effect against Gram-positive bacteria. The high correlation between the bioactivities studied and the presence of phenolic compounds was also proven, which meets consumer expectations about their health and well-being. Overall, the underexplored parts of *C. macrocarpa* presented high intrinsic bioactive properties, such as antioxidant, cytotoxic, anti-inflammatory, and antimicrobial activities, potentiated by the presence of phenolic compounds. The added value of this plant can lead to its application in several industries, with different outputs.

## Figures and Tables

**Table 1:** Retention time (Rt), wavelengths of maximum absorption in the visible region (λ_max_), mass spectral data, and tentative identification of the phenolic compounds present in the hydroethanolic extracts of *C. macrocarpa* leaves, stems, and flowers.

Peak	Rt (min)	λmax (nm)	[M − H]^−^ (*m*/*z*)	MS^2^ (*m*/*z*)	Tentative Identification
**1**	4.65	324	353	191(100),179(45),161(15),135(10)	3-*O*-Caffeolyquinic acid
**2**	5.16	310	337	191(100),173(3),161(5)	*cis* 3-*p*-Coumaroylquinic acid
**3**	5.21	284	359	239(95),197(100),181(6),153(10),137(5)	Syringic acid hexoside
**4**	5.34	290	337	191(100),173(5),161(5)	*trans* 3-*p*-Coumaroylquinic acid
**5**	6.31	287	353	191(32),173(100),161(5),135(5)	*cis* 4-*O*-Caffeolyquinic acid
**6**	6.8	325	353	191(75),173(100),161(12),135(5)	*trans* 4-*O*-Caffeolyquinic acid
**7**	7.35	281	577	425(100),289(13)	Type B (epi)catechin dimer
**8**	8.1	325	353	191(100),179(35),161(5),135(5)	5-*O*-Caffeolyquinic acid *
**9**	9.01	280	577	425(100),289(19)	Type B (epi)catechin dimer
**10**	9.45	266,347	755	593(20),285(100)	Kaempherol-*O*-hexoside-*O*-rutinoside
**11**	10.26	280	865	451(14),425(16),407(12),289(11)	Type B (epi)catechin trimer
**12**	11.47	280	1153	865(78),577(35),575(43),289(5)	Type B (epi)catechintetramer
**13**	10.77	284	337	191(100),173(5),161(5)	*cis* 5-*p*-Coumaroylquinic acid
**14**	11.49	280	1153	865(82),577(24),575(36),289(5)	Type B (epi)catechin tetramer
**15**	12.15	310	337	191(100),173(3),161(3)	*trans* 5-*p*-Coumaroylquinic acid
**16**	12.41	280	865	451(15),425(13),407(17),289(7)	Type B (epi)catechin trimer
**17**	12.9	280	863	711(26),573(61),451(12),411(5),289(22)	Type A (epi)catechin trimer
**18**	13.1	267,347	739	593(100),285(25)	Kaempferol-*O*-deoxyhexoside-*O*-deoxyhexosyl-hexoside isomer 1
**19**	13.35	280	1153	865(54),577(23),575(24),289(5)	Type B (epi)catechin tetramer
**20**	13.96	265,352	755	609(100),301(25)	Quercetin-*O*-deoxyhexoside-*O*-deoxyhexosyl-hexoside
**21**	14.07	266,357	739	593(100),285(25)	Kaempferol-*O*-deoxyhexoside-*O*-deoxyhexosyl-hexoside isomer 2
**22**	14.7	257,352	609	301(100)	Quercetin-*O*-deoxyhexosyl-hexoside isomer 1
**23**	15.62	280,339	739	285(100)	Kaempferol-*O*-di-deoxyhexoside-hexoside
**24**	15.95	271,344	575	285(100)	Acetylkaempherol-*O*-malonylhexoside
**25**	16.09	266,357	739	593(100),285(25)	Kaempferol-*O*-deoxyhexoside-*O*-deoxyhexosyl-hexoside isomer 3
**26**	16.39	257,348	739	593(100),285(25)	Kaempferol-*O*-deoxyhexoside-*O*-deoxyhexosyl-hexoside isomer 4
**27**	16.88	266,352	609	301(100)	Quercetin-*O*-deoxyhexosyl-hexoside isomer 2
**28**	17.1	257,354	609	301(100)	Quercetin-3-*O*-rutinoside *
**29**	19.17	266,346	593	285(100)	Kaempherol-*O*-deoxyhexosyl-hexoside
**30**	20.32	266,346	593	285(100)	Kaempherol-3-*O*-rutinoside *

* Compounds identified and quantified according to their chromatographic characteristics by comparison to those obtained with standard compounds.

**Table 2:** Quantification (mg/g of extract) of the phenolic compounds present in the hydroethanolic extracts of *C. macrocarpa* leaves, stems, and flowers.

Peak	Leaves	Stems	Flowers
**1**	nd	0.49 ± 0.01	nd
**2**	0.25 ± 0.01	nd	nd
**3**	nd	0.34 ± 0.01	nd
**4**	0.34 ± 0.01 ^b^	nd	0.6 ± 0.01 ^a^
**5**	0.26 ± 0.004 ^c^	0.61 ± 0.02 ^b^	3.1 ± 0.1 ^a^
**6**	0.5 ± 0.02 ^b^	2.28 ± 0.02 ^a^	nd
**7**	2 ± 0.1 ^b^	6.4 ± 0.2 ^a^	nd
**8**	nd	nd	0.17 ± 0.01
**9**	nd	2.88 ± 0.01	nd
**10**	nd	nd	0.48 ± 0.02
**11**	1.9 ± 0.1 ^b^	6.09 ± 0.02 ^a^	nd
**12**	2.9 ± 0.1	nd	nd
**13**	nd	0.97 ± 0.01 ^b^	1.21 ± 0.02 ^a^
**14**	nd	7.3 ± 0.2	nd
**15**	nd	nd	0.49 ± 0.02
**16**	1.9 ± 0.1 ^b^	3.7 ± 0.1 ^a^	nd
**17**	1.57 ± 0.05 ^b^	4.9 ± 0.2 ^a^	nd
**18**	nd	nd	tr
**19**	2.2 ± 0.1 ^b^	3.9 ± 0.1 ^a^	nd
**20**	1.79 ± 0.03	1.03 ± 0.01	nd
**21**	nd	nd	tr
**22**	nd	nd	tr
**23**	1.03 ± 0.01	nd	nd
**24**	1.1 ± 0.03	nd	nd
**25**	nd	nd	tr
**26**	nd	nd	1.6 ± 0.1
**27**	tr	nd	nd
**28**	1.86 ± 0.04 ^b^	2.6 ± 0.1 ^a^	1.48 ± 0.01 ^c^
**29**	nd	nd	tr
**30**	tr	nd	1.73 ± 0.03
**TPA**	1.35 ± 0.04 ^c^	4.68 ± 0.01 ^b^	5.5 ± 0.1 ^a^
**TF3O**	12.5 ± 0.2 ^b^	35.1 ± 0.2 ^a^	nd
**TF**	5.7 ± 0.1 ^a^	3.6 ± 0.1 ^c^	5.3 ± 0.1 ^b^
**TPC**	19.6 ± 0.3 ^b^	43.4 ± 0.1 ^a^	10.8 ± 0.2 ^c^

tr—traces; nd—not detected. Standard calibration curves: caffeic acid (y = 168823x − 161172, R^2^ = 0.9939; peaks **1**, **5**, **6**, and **8**); catechin (y = 84950x − 23200, R^2^ = 0.9999; peaks **7**, **9**, **11**, **12**, **14**, **16**, **17**, and **19**); *p*-coumaric acid (y = 301950x + 6966.7, R^2^ = 0.9999; peaks **2**, **4**, **13**, and **15**); quercetin-3-*O*-glucoside (y = 34843x − 160173, R^2^ = 0.9998; peaks **20**, **21**, **24**, and **25**); quercetin-3-*O*-rutinoside (y = 13343x + 76751, R^2^ = 0.9998; peaks **10**, **18**, **22**, **23**, **26**, **27**, **28**, **29**, **30**, and **31**). **TPA**—total phenolic acids; **TF3O**—total flavan-3-ols; **TF**—total flavonols; **TPC**—total phenolic compounds.

**Table 3:** Antioxidant, cytotoxic, anti-inflammatory, and antibacterial activities of hydroethanolic extracts of *C. macrocarpa* leaves, stems, and flowers, and their correlation with the families of the phenolic compounds identified (mean ± SD).

	Leaves	Stems	Flowers	Correlation Factor *r*^2^			
				TPA	TF3O	TF	TPC
**Antioxidant activity EC_50_ values (μg/mL) ^A^**							
DPPH scavenging activity	26 ± 1 ^b^	281 ± 1 ^a^	223 ± 6 ^a^	0.887	*w*/*n*	0.862	m
Reducing power	36 ± 1 ^b^	33 ± 1 ^a^	279 ± 4 ^b^	*w*/*n*	0.940	*w*/*n*	0.995
β-carotene bleaching inhibition	300 ± 1 ^b^	270 ± 10 ^b^	1107 ± 47 ^a^	m	0.781	0.719	m
TBARS inhibition	15.4 ± 0.1 ^b^	12.1 ± 0.1 ^c^	92.5 ± 0.1 ^a^	m	0.794	0.718	m
**Cytotoxicity GI_50_ values (μg/mL) ^B^**							
MCF-7 (breast carcinoma)	167 ± 2 ^a^	70.38 ± 0.03 ^c^	95.25 ± 0.01 ^b^	0.862	*w*/*n*	0.846	m
NCI-H460 (non-small cell lung carcinoma)	120 ± 1 ^a^	58.7 ± 0.2 ^c^	68 ± 1 ^b^	0.911	*w*/*n*	0.898	m
HeLa (cervical carcinoma)	101 ± 1 ^a^	52.1 ± 0.3 ^c^	75 ± 1 ^b^	0.721	m	m	0.781
HepG2 (hepatocellular carcinoma)	152 ± 3 ^a^	89 ± 1 ^b^	>400	0.943	*w*/*n*	0.953	*w*/*n*
PLP2 (non-tumour porcine liver primary cells)	>400	>400	>400	-	-	-	-
**Anti-inflammatory activity IC_50_ values (μg/mL) ^C^**							
NO production	179 ± 6 ^c^	208 ± 9 ^a^	196 ± 4 ^b^	m	*w*/*n*	*w*/*n*	*w*/*n*
**Antibacterial activity MIC values (mg/mL)**							
**Gram-negative bacteria**							
*Escherichia coli* ^D^	5	10	10	0.961	*w*/*n*	0.952	*w*/*n*
*Escherichia coli* ESBL ^E^	20	20	20	-	-	-	-
*Klebsiella pneumoniae* ^D^	>20	>20	>20	-	-	-	-
*Klebsiella pneumoniae* ESBL ^D^	>20	>20	>20	-	-	-	-
*Morganella morganii* ^D^	10	10	20	0.720	0.772	0.774	*w*/*n*
*Pseudomonas aeruginosa*	20	20	20	-	-	-	-
**Gram-positive bacteria**							
*Enterococcus faecalis* ^F^	1.25	1.25	5	0.720	0.772	0.774	*w*/*n*
*Listeria monocytogenes* ^G^	2.5	0.625	20	m	0.825	m	m
MRSA *^,F^	2.5	2.5	20	0.720	0.772	0.742	m
MSSA ^F^	5	2.5	10	*w*/*n*	0.938	m	0.852

^A^ Trolox EC_50_ values: 43.03 ± 1.71 μg/mL (DDPH), 29.62 ± 3.15 μg/mL (reducing power), 2.63 ± 0.14 μg/mL (β-carotene bleaching inhibition), and 3.73 ± 1.9 μg/mL (TBARS inhibition); ^B^ Ellipticine GI_50_ value: 0.91 ± 0.04 μg/mL (MCF-7), 1.03 ± 0.09 μg/mL (NCI-H460), 1.91 ± 0.06 μg/mL (HeLa), 1.1 ± 0.2 μg/mL (HepG2), and 3.2 ± 0.7 μg/mL (PLP2); ^C^ Dexamethaxone EC_50_ value: 16 ± 1 μg/mL. S—susceptible; I—intermediate; R—resistant. This classification was made according to the interpretative breakpoints suggested by Clinical and Laboratory Standards Institute (CLSI) and the European Committee on Antimicrobial Susceptibility Testing (EUCAST): ^D^ Amoxicillin/Clavulanic acid (*E. coli* ≤ 8/4, S; *K. pneumoniae* ≤ 8/4, S; *K. pneumoniae* ESBL ≥ 32, R; *M. morganii* > 16/8, R); ^E^ Amikacin (*E. coli* ESBL 16, I); ^F^ Vancomycin (*E. faecalis*, MRSA, and MSSA ≤ 2, S); ^G^ Ampicillin (*L. monocytogenes* ≤ 0.2, S). In each row and for the different extraction procedure, different letters mean significant differences (*p* < 0.05). Data shown on correlation factor only considered the strong and very strong correlations ((0.7–0.9) and > 0.9, respectively); m—moderate correlations ((0.5–0.7); *w*/*n*—weak and negligible correlations ((0.3–0.5) and (0–0.3), respectively). MIC—minimum inhibitory concentration.
